# Supplementary material for: MOIRE: A software package for the estimation of allele frequencies and effective multiplicity of infection from polyallelic data
Source: bioRxiv. 2023 Oct 5:2023.10.03.560769. Preprint. [Version 1] doi: 10.1101/2023.10.03.560769 (PMC10592951; doi:10.1101/2023.10.03.560769)
Supplement: Supplementary file 1 [file NIHPP2023.10.03.560769v1-supplement-1.pdf]

6 Supplementary Material

6.1 Supplementary Material: Alternative Latent Genetic State Formulation

While the formulation of latent genetic state as a draw from a multinomial distribution that is then masked into a binary vector indicating presence or absence is intuitive, it is a computationally inefficient approach as we must enumerate all possible arrangements that are compatible with the latent genetic state. We can

bioRxiv preprint doi: https://doi.org/10.1101/2023.10.03.560769; this version posted October 5, 2023. The copyright holder for this preprint (which was not certified by peer review) is the author/funder, who has granted bioRxiv a license to display the preprint in perpetuity. It is made available under aCC-BY 4.0 International license.

instead consider an alternative but equivalent formulation. As before, we are interested in calculating the probability of the latent genetic state, $P(Y_{ij}|m = k, \mu_i, \pi_j)$. Let O_{ij} be the set of indices of alleles that are observed in sample i at locus j. For example, if $Y_{ij} = (0, 1, 1, 0)$ then $O_{ij} = \{2, 3\}$. Let $\pi(O_{ij})$ equal the sum of allele frequencies indexed by O_{ij}, and $n = \mu_i - k$, the number of unrelated strains present. We can then write the probability of the latent genetic state as

$$P(Y_{ij}|m = k, \mu_i, \pi_j) = P(O_{ij}|n, \pi(O_{ij}))\pi(O_{ij})^n \tag{9}$$

where $P(O_{ij}|n, \pi(O_{ij}))$ is the probability that each allele indexed by O_{ij} is present at least once after k draws, conditional on that all draws are from the set of alleles indexed by O_{ij}, and $\pi(O_{ij})^n$ is the probability that all n alleles come from the set of alleles indexed by O_{ij}. The probability of every allele being present at least once is equal to 1 minus the probability that any allele is not present, which can be calculated via the inclusion exclusion principle:

$$P(O_{ij}|n, \pi(O_{ij})) = 1 - \sum_{S \subseteq O_{ij}} (-1)^{|S|-1}(1 - \sum_{a \in S} \frac{\pi_{ja}}{\pi(O_{ij})})^n \tag{10}$$

where S is a subset of O_{ij} and $|S|$ is the cardinality of S. This formulation is computationally more efficient, though less intuitive, by allowing us to calculate the probability of the latent genetic state without directly enumerating all possible arrangements of alleles that are compatible with the latent genetic state.

6.2 Supplementary Material: Sampling and Inference

Error Rates

Sample specific false positive and false negative rates were randomly initialized to a value between 0 and 0.1. False positive and false negative rates were constrained to be between 0 and 2, so sampling was conducted after transforming to an unconstrained space to improve efficiency. Proposals were sampled from a normal distribution centered around the transformed current value with a standard deviation that was adapted during burnin to achieve an acceptance rate of 0.23 with proposals accepted according to a Metropolis-Hastings acceptance probability [27, 28]. Priors were uniform between 0 and 1.

Within-host Relatedness

Within-host relatedness was randomly initialized uniformly between 0 and 1 for each sample and constrained to be between 0 and 1, so sampling was conducted after transforming to an unconstrained space to improve efficiency. Proposals were sampled from a normal distribution centered around the transformed current value with a standard deviation that was adapted during burnin to achieve an acceptance rate of 0.23 with proposals accepted according to a Metropolis-Hastings acceptance probability. Priors were uniform between 0 and 1, reflecting our lack of prior knowledge about within-host relatedness.

MOI and Latent Genotypes

Sample specific MOIs were randomly initialized to a value between the maximum number of observed alleles ± 3 without exceeding specified constraints. Sample specific MOIs were constrained to be between 0 and 40. Latent genotypes were initialized to the observed genotype. Proposals for sample MOIs were generated by sampling from a symmetric distribution centered around the current value. Proposals that exceeded the constraints were rejected. Simultaneously, latent genotypes for each locus were sampled by randomly sampling the number of false positives and false negatives present, while ensuring at least one allele being a true positive, and the total number of true positives not exceeding the proposed MOI. The number of false positives and false negatives were sampled from a binomial distribution with the number of trials selected to satisfy the previously described constraints, and the probability of success equal to the false positive and false negative rates at that step, respectively. The alleles that were false positives or false negatives were

bioRxiv preprint doi: https://doi.org/10.1101/2023.10.03.560769; this version posted October 5, 2023. The copyright holder for this preprint (which was not certified by peer review) is the author/funder, who has granted bioRxiv a license to display the preprint in perpetuity. It is made available under aCC-BY 4.0 International license.

randomly selected from the observed genotype. Proposals were accepted according to a Metropolis-Hastings acceptance probability.

Allele Frequencies

Allele frequencies were randomly initialized on the unit simplex. Allele frequencies were then sampled according to the self adjusting logit transform proposal, also known as the SALT sampler [29] and accepted according to a Metropolis-Hastings acceptance probability.

Mean MOI

The mean MOI was randomly initialized to a random draw from the prior distribution. The mean MOI was constrained to be between 0 and 40, so sampling was conducted after transforming to an unconstrained space to improve efficiency. Proposals were sampled from a normal distribution centered around the transformed current value with a standard deviation that was adapted during burnin to achieve an acceptance rate of 0.23 with proposalas accepted according to a Metropolis-Hastings acceptance probability. The hyperprior on the mean MOI was a gamma distribution with shape and scale parameters of 0.1 and 10 respectively, reflecting our assumptions around low mean MOI.

Metropolis Coupled Markov Chain Monte Carlo

It may be the case that the posterior distribution is multimodal, and that the Markov chain may get stuck in a local maxima, leading to poor mixing. To address this, we use Metropolis Coupled Markov Chain Monte Carlo (MC^3), also sometimes referred to as parallel tempering, or replica exchange MCMC sampling [30]. We provide a fully parallelized implementation, allowing for the full utilization of modern high performance computing clusters or multicore desktop machines. We also leverage a non-reversible algorithm with an adaptive temperature gradient as described by [31] to further improve mixing and reduce tuning required by the user.

6.3 Supplementary Material: Effective MOI

Consider an infection with n distinct unrelated strains drawn from the background population. Let X be the observed genotype at a single locus with L possible alleles and population frequencies π, where every allele has a non-zero frequency in the population, and D be the number of distinct alleles observed at the locus

$$D = \sum_{i=1}^{L} \mathbb{I}(X_i = 1) \tag{11}$$

where $X_i = 1$ if allele i is observed. By linearity of expectation, the expected number of distinct alleles (EDA) is

$$\begin{aligned}
\mathbb{E}[D] &= \sum_{i=1}^{L} \mathbb{E}[\mathbb{I}(X_i = 1)] && (12)\\
&= \sum_{i=1}^{L} \mathbb{P}(X_i = 1) && (13)\\
&= \sum_{i=1}^{L} 1 - (1 - \pi_i)^n && (14)\\
& && (15)
\end{aligned}$$

bioRxiv preprint doi: https://doi.org/10.1101/2023.10.03.560769; this version posted October 5, 2023. The copyright holder for this preprint (which was not certified by peer review) is the author/funder, who has granted bioRxiv a license to display the preprint in perpetuity. It is made available under aCC-BY 4.0 International license.

where $(1-\pi_i)^n$ is the probability that allele i is not observed in any of the n strains.

We note that the EDA is a metric that is dependent on MOI and allele frequencies, and is itself an interesting quantity. In the special case of $n = 2$, it is equivalent to the heterozygosity of the locus after subtracting 1. When $n > 2$, the EDA is no longer necessarily equivalent. Unsurprisingly, the EDA for a fixed n and parameterized by allele frequencies is maximized under the same conditions as the heterozygosity (when all alleles are equally likely to be observed). However, loci with differing allele frequencies (in distribution and cardinality) may have the same heterozygosity but different EDAs. This suggests that heterozygosity may be an imperfect metric of diversity in the context of mixed infections. For a fixed MOI, a higher EDA indicates greater information capacity in the locus, and thus higher precision as a tool for estimating parameters. A locus with higher EDA should be preferred when considering loci for genotyping panel development.

We also note that the EDA of a locus may not be strictly larger than the EDA of another locus across MOI, suggesting that genetic loci are more powerful for statistical purposes under limited ranges. Because of this, EDA should be considered in the context of the population distribution of MOI. In principle, it would be possible to marginalize over an estimate of the population distribution of MOI to obtain a generalized estimate of EDA that is weighted by the probability of observing a given MOI, providing a metric that is targeted to the population of interest.

The EDA also provides a natural approach to defining effective MOI. Consider an idealized locus with a heterozygosity of 1, meaning that every allele drawn from the population is unique. Practically, this is an impossibility, however we may approximate such a locus by considering the limit as the number of alleles goes to infinity and occur with equal probability. Let $L \to \infty$ and $\pi_i = \frac{1}{L}$, then the EDA for an infection with n distinct strains is

$$\lim_{L\to\infty} E[D] = \lim_{L\to\infty} \sum_{i=1}^{L} 1 - (1 - \frac{1}{L})^n \tag{16}$$

$$= \lim_{L\to\infty} L - L(\frac{L-1}{L})^n \tag{17}$$

$$= n \tag{18}$$

As expected, in the limit of an infinitely diverse locus, the EDA is equal to the MOI. We now consider the EDA for an infinitely diverse locus in an infection with within-host relatedness r. While MOI remains a fixed quantity, the number of related strains is now a random variable distributed as a binomial with parameters $n-1$ and r. We define the effective MOI (eMOI) as the expected number of distinct alleles observed in an infection with n distinct strains and within-host relatedness r at a locus with infinite diversity. By construction, only unrelated strains contribute to the EDA, thus the eMOI is simply the EDA marginalized over the distribution of unrelated strains in an infection with n distinct strains and within-host relatedness r

$$\text{eMOI} = \sum_{k=0}^{n-1} \binom{n-1}{k} r^k (1-r)^{n-1-k}(n-k) \tag{19}$$

$$= 1 + (1-r)(n-1) \tag{20}$$

As r goes to zero, this quantity approaches the MOI, and as r goes to one, this quantity approaches 1, recovering a natural measure of within-host diversity that is sensitive to both MOI and within-host relatedness.

bioRxiv preprint doi: https://doi.org/10.1101/2023.10.03.560769; this version posted October 5, 2023. The copyright holder for this preprint (which was not certified by peer review) is the author/funder, who has granted bioRxiv a license to display the preprint in perpetuity. It is made available under aCC-BY 4.0 International license.

6.4 Supplementary Material: Regional Populations

In order to assess potential real world performance of MOIRE, we simulated genotyping data from 12 regional populations parameterized by the MalariaGEN Pf7 dataset [19]. Marginal allele frequencies were estimated within each region for each genotyping panel from whole genome sequencing data available from the Pf7 dataset. Alleles within regions with a frequency below 1% were excluded from microhaplotype panels to avoid excessive computational burden when running MOIRE. Uninformative loci (only 1 allele observed) were removed for each region. Interquartile ranges of the number of alleles observed and the number of loci in each panel are summarized in Table S1. Summaries of panel diversity, evaluated by calculating the expected number of distinct alleles from 2 to 10 possible strains, are provided in figure S3. We note that not all unique alleles included in the simulation may be reliably genotypable, e.g. homopolymers and tandem repeats, depending on the fidelity of amplification and sequencing. Simulations may provide an optimistic estimate of performance.

**Region**	AMPLseq	MaD^{4}HatTeR	AmpliSeq
Central Africa	3-8 (n=121)	4-7 (n=165)	2-10 (n=158)
Central West Africa	3-8 (n=121)	3-7 (n=164)	2-10 (n=155)
East Africa	3-8 (n=122)	4-8 (n=165)	2-11 (n=156)
Papua New Guinea	2-4 (n=115)	2-5 (n=151)	2-6 (n=148)
South Africa	3-9 (n=125)	4-8 (n=165)	2-7 (n=161)
South America - Central	2-3 (n=106)	2-3 (n=141)	2-4 (n=128)
South America - North	2-3 (n=107)	2-3 (n=136)	2-3 (n=114)
South Asia	3-7 (n=121)	3-7 (n=165)	2-9 (n=155)
South East Africa	3-8 (n=119)	4-7 (n=165)	2-11 (n=153)
South East Asia - East	2-5 (n=106)	2-5 (n=137)	2-7 (n=129)
South East Asia - West	2-5 (n=110)	2-4 (n=147)	2-5 (n=148)
West Africa	2-8 (n=122)	3-7 (n=164)	2-11 (n=149)

Table S1. Interquartile range (IQR) of the number of alleles per locus for each region and genotyping panel. Number of loci included in each panel is indicated in parentheses.

bioRxiv preprint doi: https://doi.org/10.1101/2023.10.03.560769; this version posted October 5, 2023. The copyright holder for this preprint (which was not certified by peer review) is the author/funder, who has granted bioRxiv a license to display the preprint in perpetuity. It is made available under aCC-BY 4.0 International license.

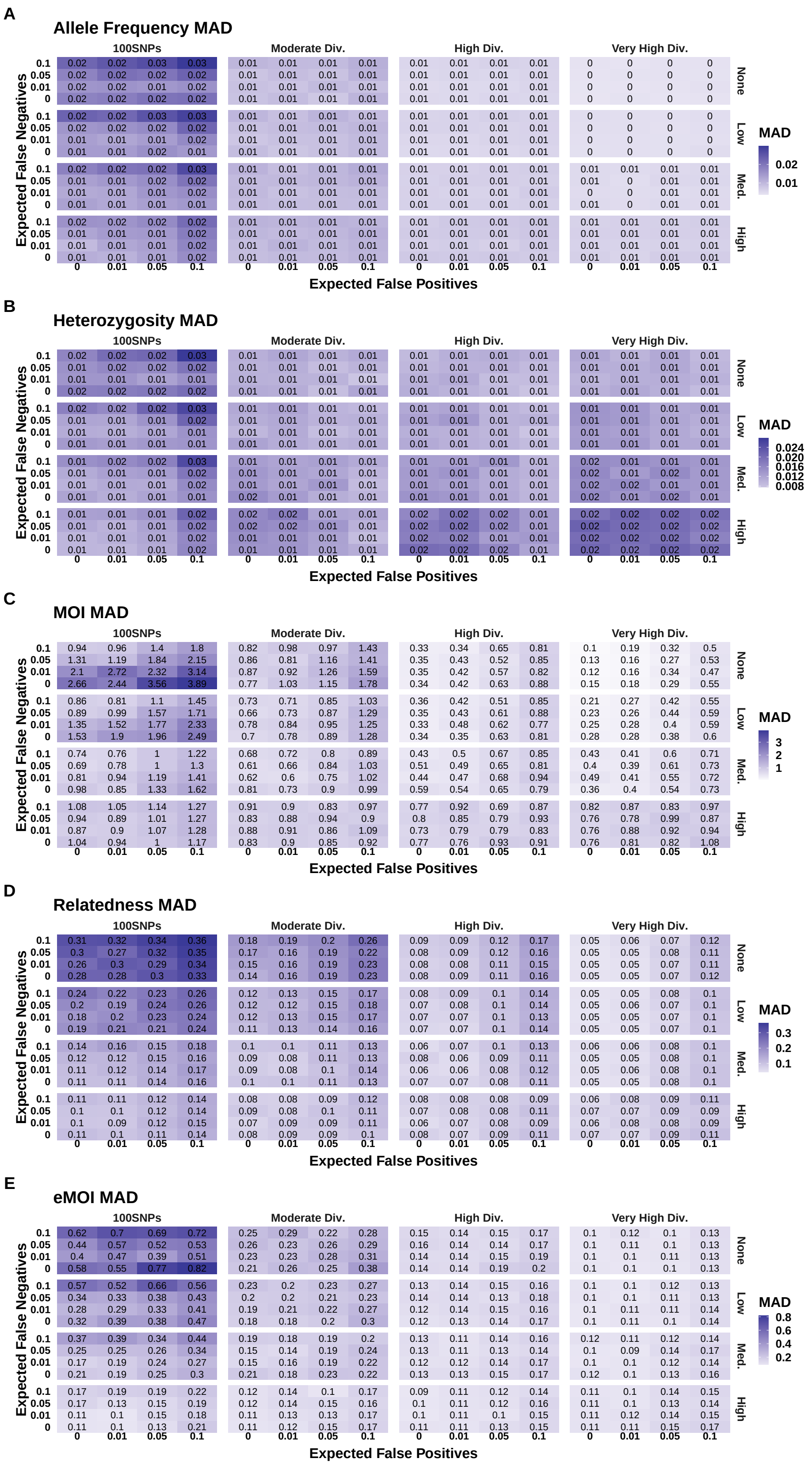

Figure S1. Mean absolute deviation (MAD) of parameter estimates by MOIRE across panels of varying genetic diversity and stratified by population levels of within-host relatedness.

bioRxiv preprint doi: https://doi.org/10.1101/2023.10.03.560769; this version posted October 5, 2023. The copyright holder for this preprint (which was not certified by peer review) is the author/funder, who has granted bioRxiv a license to display the preprint in perpetuity. It is made available under aCC-BY 4.0 International license.

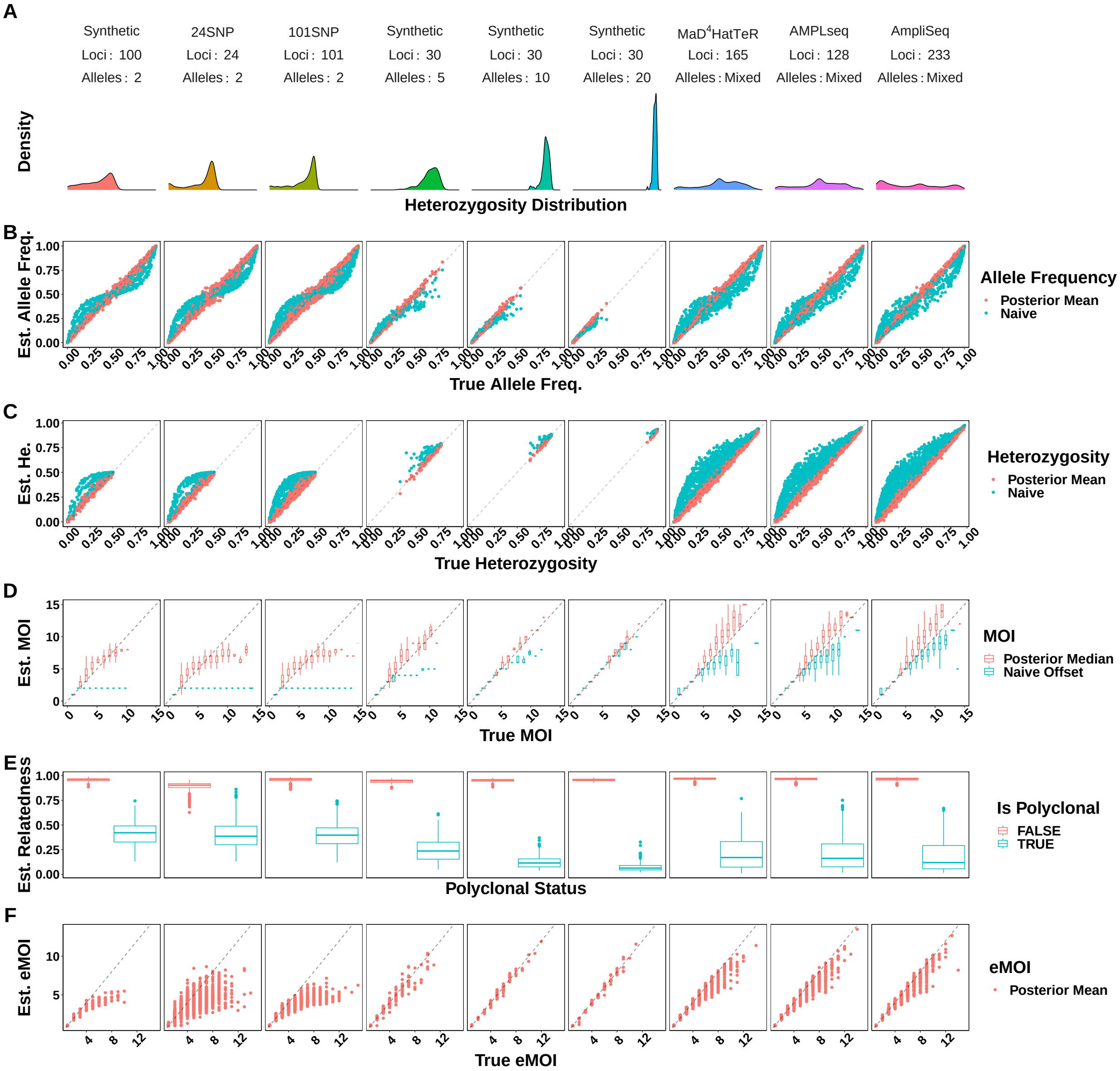

Figure S2. Attempting to estimate relatedness in the absence of relatedness did not introduce bias into parameters of interest such as MOI, heterozygosity, and allele frequencies. Simulations were pooled across mean MOIs. False positive and false negatives rates were fixed to 0.01 and 0.1 respectively.

bioRxiv preprint doi: https://doi.org/10.1101/2023.10.03.560769; this version posted October 5, 2023. The copyright holder for this preprint (which was not certified by peer review) is the author/funder, who has granted bioRxiv a license to display the preprint in perpetuity. It is made available under aCC-BY 4.0 International license.

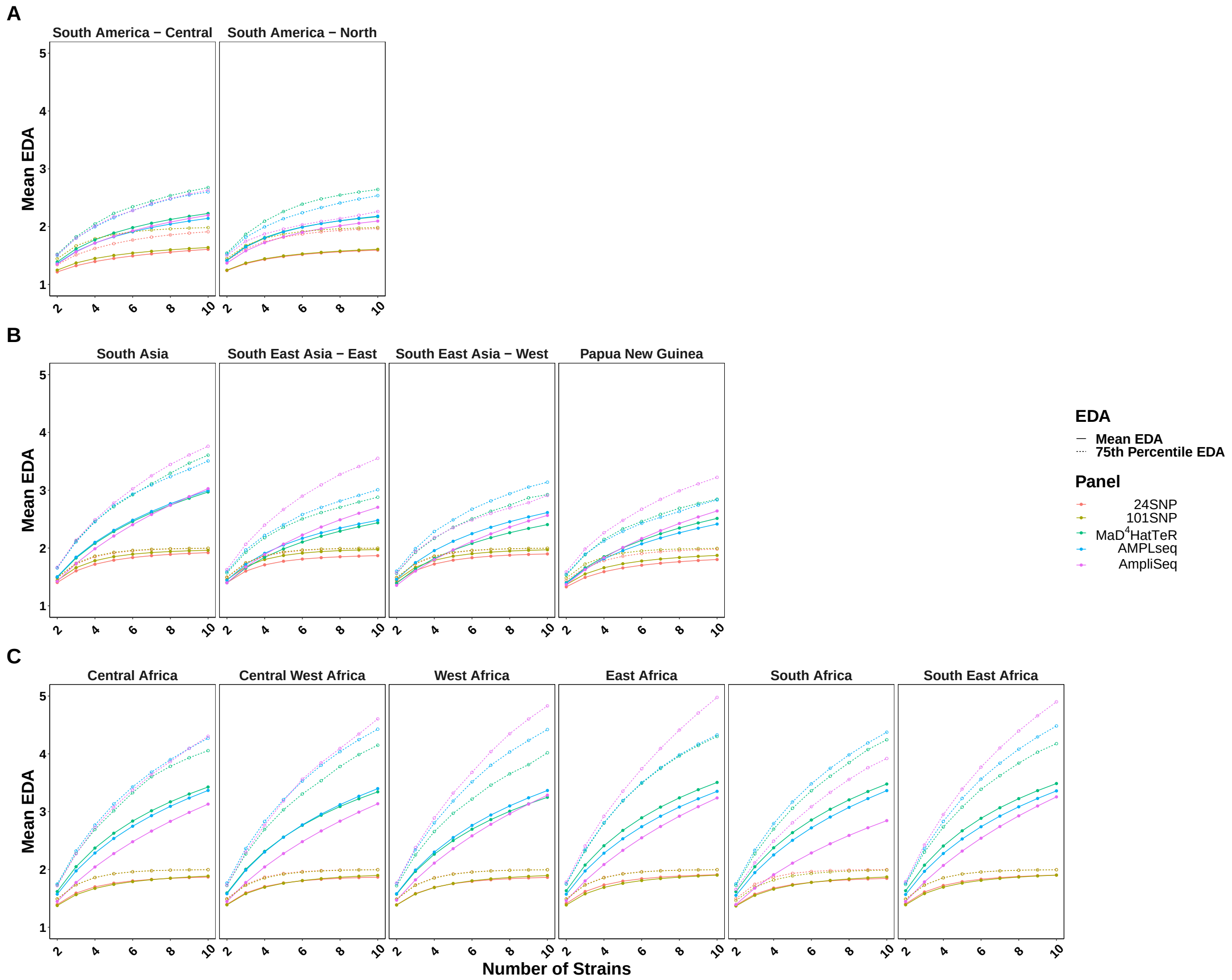

Figure S3. Mean EDA across 12 regional populations for 5 selected genotyping panels. Marginal allele frequencies within each regional population were estimated from the MalariaGEN Pf7 dataset. We then evaluated the expected number of distinct alleles for each locus within each regional population for each genotyping panel. The solid lines indicate the mean EDA across loci within each regional population, and the dashed lines indicate the 75th percentile. We note that heterozygosity for each panel is equal to the EDA minus 1 when the number of strains is 2.
